# One-year results from the Assessing MICRO-vascular resistances *via* IMR to predict outcome in ST-elevation myocardial infarction patients with multivessel disease undergoing primary PCI (AMICRO) trial

**DOI:** 10.3389/fcvm.2022.1051174

**Published:** 2022-12-02

**Authors:** Massimo Fineschi, Edoardo Verna, Alberto Barioli, Giuseppe Mezzapelle, Davide Bartolini, Giovanni Turiano, Vincenzo Guiducci, Antonio Manari, Katya Lucarelli, Lucia Uguccioni, Alessandra Repetto, Giuseppe Tarantini

**Affiliations:** ^1^Policlinico Santa Maria alle Scotte, Siena, SI, Italy; ^2^Ospedale di Circolo e Fondazione Macchi, Università dell’Insubria, Varese, VA, Italy; ^3^Ospedale Ca’ Foncello, Treviso, TV, Italy; ^4^Azienda Ospedaliera Universitaria, Padova, PD, Italy; ^5^Ospedale Giovanni Paolo II, Sciacca, AG, Italy; ^6^Azienda Ospedaliera Villa Scassi, Genoa, Italy; ^7^Ospedale di Conegliano, Conegliano, TV, Italy; ^8^Azienda USL-IRCCS, Reggio Emilia, RE, Italy; ^9^Ospedale Generale Regionale F. Miulli, Acquaviva delle Fonti, BA, Italy; ^10^Ospedali Riuniti Marche Nord, Pesaro, PU, Italy; ^11^Fondazione IRCCS Policlinico S. Matteo, Pavia, PV, Italy

**Keywords:** STEMI, multivessel disease (MVD), microvascular resistance, índex of microvascular resistance (IMR), fractional flow reserve (FFR)

## Abstract

**Background:**

In ST-elevation myocardial infarction (STEMI) patients undergoing primary percutaneous coronary angioplasty (PPCI) the index of microcirculatory resistance (IMR) correlates to the extent of myocardial damage and left ventricular (LV) function recovery. Data on the IMR time-course and impact on clinical outcome in STEMI patients with multi-vessel disease (MVD) are scarce.

**Aims:**

We designed a prospective, multicenter clinical trial to assess the infarct-related artery (IRA)-IMR in STEMI patients with MVD undergoing PPCI and to explore its potential in relationship with outcome and LV remodeling.

**Methods:**

The study enrolled 242 STEMI patients with MVD. Both fractional flow reserve (FFR) and IMR of the IRA were assessed after successful PPCI. Then, FFR/IMR measurements were repeated in the IRA at a staged angiography, and FFR-guided angioplasty was performed in non-IRA lesions. The primary endpoint was the composite of cardiovascular death, re-infarction, re-hospitalization for heart failure, resuscitation or appropriate ICD shock at 1-year follow-up.

**Results:**

A significant improvement of IRA-IMR values (from 47.9 to 34.2, *p* < 0.0001) was observed early after PPCI. Staged FFR-guided angioplasty was performed in 102 non-IRA lesions. We failed to find a correlation between IRA-IMR, clinical events and LV remodeling. Notwithstanding, in patients with anterior STEMI an inverse correlation between initial IMR values and LV function at follow-up was observed.

**Conclusion:**

After successful PPCI, a significant proportion of patients with STEMI and MVD had coronary microvascular dysfunction as assessed by IMR that recovered early after reperfusion. Higher IMR values predicted lack of improvement of LV function only in anterior STEMI.

**Clinical trial registration:**

https://clinicaltrials.gov/, identifier [NCT 02325973].

## Introduction

Although primary percutaneous coronary intervention (PPCI), in comparison with thrombolysis, may guarantee a higher rate of recanalization in ST-elevation myocardial infarction (STEMI) patients, it cannot fully prevent tissue and microvascular damage, which are commonly seen and strongly related to the delay of reperfusion (1–3). Interestingly, although infarct size is a major determinant of microvascular obstruction at any given delay in treatment, both experimental and clinical studies suggest that microvascular obstruction *per se* is a stronger predictor of worse outcome and left ventricular function compared with infarct size (4–6). Myocardial reperfusion has been previously assessed by means of ST-segment resolution (7), myocardial blush grade (8), thrombolysis in myocardial infarction (TIMI) perfusion grade (9), myocardial contrast echocardiography and magnetic resonance (CMR) imaging (10, 11). In the last few decades, a method based on a pressure sensor/thermistor-tipped guidewire, that allows for measurement of hyperemic distal coronary pressure and blood flow by thermodilution technique and that permit calculation of an index of microcirculatory coronary resistance (IMR) (12–16), has been introduced. The IMR appears promising in the assessment of the extent of microvascular damage. However, the extent, the time-course and the role of IMR after PPCI is less well defined. Only few studies addressed this point in patients with predominantly single-vessel disease (17–20). Nevertheless, patients with multivessel disease (MVD) may have worse prognosis after PPCI for STEMI compared to patients with single vessel disease (21, 22).

The impact of IMR on clinical outcome in PPCI patients with MVD remains unsettled. We designed a prospective, multicenter clinical trial to assess the infarct-related artery (IRA) IMR time-course in STEMI patients with MVD undergoing PPCI and to explore its relationship with outcome and LV remodeling.

## Materials and methods

### Study design

The AMICRO (“Assessing MICRO-vascular resistances *via* IMR to predict outcome in STEMI patients with multivessel disease undergoing primary PCI”) trial was a prospective, multicenter clinical study that included patients with MVD undergoing PPCI for STEMI in 11 hospitals in Italy. The study design and statistical plan has been described previously in detail (23). The study complies with the Declaration of Helsinki concerning medical research and with local legal and regulatory requirements. The trial was a St. Jude Medical (now Abbott Medical) sponsored study, and all study analyses were conducted with the assistance of Abbott. The AMICRO trial is registered with the National Institutes of Health sponsored ClinicalTrials.gov (www.clinicaltrials.gov) as study number NCT 02325973.

### Patients

Patient enrollment started on June 2013. Key eligibility criteria were as follows: (1) diagnosis of STEMI with typical chest pain and ST-segment elevation of >0.1 mV in ≥2 consecutive leads on surface ECG or left bundle-branch block; (2) hospital admission either within 12 h of symptom onset or between 12 and 24 h after onset with evidence of continuing ischemia; (3) clearly defined infarct-related culprit lesion on index angiography and evidence of MVD defined as the presence of at least one angiographically significant (>50% by visual estimation) non-culprit lesion in a non-infarct-related artery; (4) successful drug-eluting stent PPCI of the culprit lesion. Key exclusion criteria included life expectancy of less than 1-year, prior myocardial infarction on the same area, hemodynamic instability not controllable with medical therapy and/or intra-aortic balloon pumping, prior coronary artery bypass grafting (CABG), and left main coronary artery disease or indication for CABG. All patients provided written informed consent.

### Procedures

Patients were enrolled if they met all eligibility criteria. At index coronary angiogram the severity of all coronary artery lesions and the angiographic SYNTAX Score™ (SS) were reported. Percutaneous coronary intervention was performed according to the current standard of care. Deployment of a second-generation drug-eluting stent with proper stent post-dilatation was required to fix the culprit lesion. After angiographically successful PPCI, both FFR and IMR were measured in the IRA. Measurements of FFR and IMR were repeated in the IRA and performed in the non-culprit vessels at pre-discharge staged coronary angiography. After functional evaluation of all coronary artery stenoses, the “functional” SYNTAX Score™ (FSS) was calculated, and functionally significant (FFR < 0.75) non-culprit artery lesions were treated by PCI accordingly.

Twelve-lead ECG and cardiac enzyme level were recorded at the time of hospital admission. Blood samples were taken to measure in-hospital peak of cardiac enzymes [creatine-phosphokinase (CK), myocardial CK (MB-CK) and/or Troponin-I (Tn-I)] as per hospital clinical practice. Medical treatment during hospitalization and on hospital discharge was left to clinical practice of the enrolling site, guidelines and standard of care recommendations. To assess clinical status, medication and adverse events, patients were followed by hospital visit at 1 and 12 months after hospital discharge; additional 6 months visit has been done by phone. Echocardiographic evaluation of changes in LV volumes and ejection fraction (EF) from hospital discharge to 1-year follow-up visit was also performed.

### Outcomes

The primary endpoint was the composite of cardiovascular death, re-myocardial infarction, re-hospitalization for heart failure, resuscitation or appropriate ICD shock at 1 year. Secondary endpoints were the evaluation of IMR index better cut-off based on primary endpoint events, new congestive heart failure (CHF) during index hospitalization and LV remodeling, new revascularization and stent thrombosis at 1 year follow-up, and additional possible event predictors.

### Statistical analysis

Statistical analyses were performed as previously outlined in the study design publication (23). To calculate the sample size, a cut-off IMR value of 32 was assumed, as previously reported (16, 24). An incidence of events/year of 7% in the group of patients with lower IMR values and of 21% in the group with higher IMR values has been hypothesized. With an alpha error = 0.05 and a power of 85%, and on applying the 2-tailed χ2 test, the final estimated sample size was of 242 patients assuming a dropout rate of 10%. Categorical variables are reported as frequencies and relative percentages, whereas continuous variables are described by means of position indexes (mean, median) and indexes of dispersion (standard deviation, range). For any given parameter estimate, a 95% confidence interval (CI) was provided. Composite endpoints were evaluated as time to first event, whichever individual component occurred first. The Fisher’s exact test was used to evaluate the existence of associations between IMR values (above and below the median or the cut-off value of 32) and outcome. In addition, the correlation between the delta IMR, expressed as the absolute average variation [Δ = IMR(T0) – IMR(T1)] or the average percent change [Δ% = [IMR(T0) – IMR(T1)]/IMR(T0)%] of the index from time 0 (T0) to time 1 (T1), and outcomes was investigated. Continuous variables were compared using the Wilcoxon–Mann–Whitney test. A discriminant model (ROC curve) was used to determine whether there were IMR threshold values (cut-off) that allowed patients to be classified as being at increased or decreased risk of future events. For all comparisons, differences were considered statistically significant when *p* < 0.05.

## Results

**FIGURE 1:**
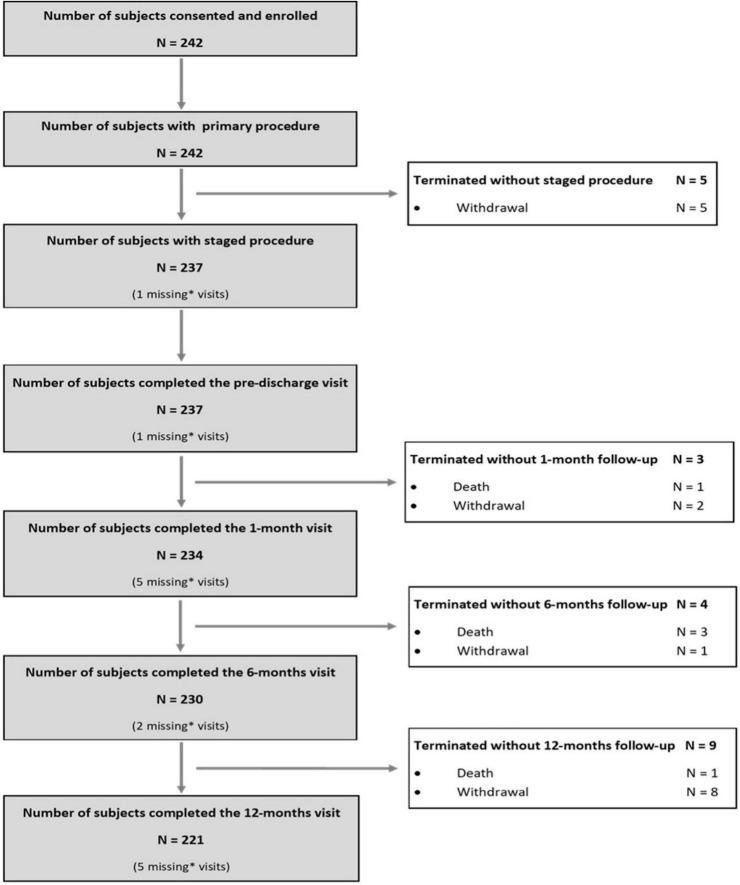
Flow chart of enrolled patients.

As per protocol, 242 subjects were enrolled in the AMICRO study between June 13, 2013 and February 14, 2017 in 11 Italian centers. Last 12 months follow-up examination was completed on March 22, 2018. 221 subjects (91.3%) completed the study participation; the remaining patients were withdrawn (16) or lost to 12M follow up (5) (Figure 1). Baseline clinical and angiographic characteristics of the enrolled patients are shown in Table 1. The mean age was 63.5 years, and more than three quarters of participants were male. Hypertension was present in 47.9% of the total population, 43.4% had dyslipidemia, and 15.3% diabetes. A small percentage of patients had previous MI (2.9%) or prior PCI (5.3%). At admission, 95% of patients were in Killip class I and 5% in Killip class II. All patients underwent successful PPCI of the IRA within a mean of 6 h of symptoms onset (median time = 3 h). All patients had multivessel coronary artery disease (2.7 lesions per patient), and three-vessel disease was present in 86 patients (35.5%). Mean baseline angiographic SS was 16.3 ± 5.8. LAD was the IRA in 43% of total cases.

**TABLE 1:** Baseline patient characteristics and lesions.

Baseline demographics		*n* = 242
Age, years		63.5 ± 9.9
Male, *n* (%)		207 (85.5)
**Medical history**		
None, *n* (%)		23 (9.5%)
Hypertension, *n* (%)		116 (47.9%)
Diabetes, *n* (%)		37 (15.3%)
	*Diet*	*5 (2.1%)*
	*Insulin*	*7 (2.9%)*
	*Oral treatment*	*25 (10.3%)*
Hyperlipidemia, dyslipidemia, *n* (%)		105 (43.4%)
Renal dysfunction*, *n* (%)	*With dialysis*	*0 (0%)*
	*Without dialysis*	*3 (1.2%)*
Significant alcohol intake, *n* (%)		3 (1.2%)
Smoker, *n* (%)		131 (54%)
	*Current*	*100 (41.3%)*
	*Ex*	*31 (12.8%)*
Other, *n* (%)		17 (7%)
**Cardiovascular history**		
None, *n* (%)		148 (61.2%)
Stroke, *n* (%)		3 (1.2%)
TIA, *n* (%)		5 (2.1%)
Myocardial infarction, *n* (%)		7 (2.9%)
Prior PCI, *n* (%)		13 (5.3%)
Family history of heart disease, *n* (%)		67 (27.7%)
Story of heart failure, *n* (%)		3 (1.2%)
Other, *n* (%)		12 (4.9%)
**Coronary lesions characteristics**		
SS (*n* = 242)		16.3 ± 5.8
FSS (*n* = 234)		11.9 ± 6.1
Three-vessel disease		86 (35.5%)
IRA, *n* (%) (*n* = 242)	*LAD*	*104 (43%)*
	*RCA*	*100 (41.3%)*
	*LCX*	*38 (15.7%)*
Non-IRA stenosis (>50%), *n* (%) (*n* = 430)	*LAD*	*171 (39.7%)*
	*RCA*	*103 (23.9%)*
	*LCX*	*156 (36.2%)*

Values are % (n) or mean ± SD.

*Defined as an estimated glomerular filtration rate (eGFR) of less than 60 ml/min/1.73 m^2^. TIA, transient ischemic attach; PCI, percutaneous coronary angioplasty; SS, syntax score; FSS, functional syntax score; IRA, infarct related artery; LAD, left anterior descending artery; RCA, right coronary artery; LCX, left circumflex artery.

### Index of microcirculatory resistance measurements

After successful PPCI, IMR was measured in 236 (97.5%) IRA lesions with a mean value of 47.9 ± 42.7. IMR was found to be higher than the pre-defined cut-off of 32 in 127 patients (53.8% of the total population). Staged angiography and physiologic evaluation were performed in 236 subjects (97.5%) within 5.2 ± 3.7 days from index procedure. Infarct-related artery IMR values significantly decreased (from 47.9 ± 42.7 to 34.2 ± 28.5, *p* < 0.0001) from index to staged procedure.

### Fractional flow reserve measurements

Fractional flow reserve (FFR) evaluation was performed in 238 (98.3%) IRA lesions after PPCI. Mean FFR was 0.92 ± 0.06 and did not change when measured at staged procedure (FFR at staged procedure 0.92 ± 0.06, *p* = NS). Physiologic evaluation of non-culprit lesions was also performed at staged procedure. Among 430 non-IRA lesions (angiographically >50% by visual estimation, mean angiographic stenosis 73.2% ± 14.5), 309 were tested with FFR and only 113 (36.6%) were significant (FFR was lower than 0.75 in 78 patients and between 0.75 and 0.80 in 35 patients). Of these, 102 (90%) underwent staged FFR-guided PCI. Functional assessment of non-IRA lesions was not performed in the remaining 121 lesions because of technical issues (i.e., vessel anatomy, lesion distality, patient non-compliance; Supplementary Figure 1 in Supplementary material). Mean syntax score after functional stenosis evaluation (FSS) was significantly lower than the baseline angiographic SS (FSS 11.9 ± 6.1 vs. SS 16.3 ± 5.8; *p* < 0.001).

### Primary endpoint results and correlation between index of microcirculatory resistance and outcomes

Twenty patients experienced cardiac events during the follow-up period: there were 4 cardiac deaths (1.7%), 2 re-hospitalizations for CHF, 2 resuscitations by ICD appropriate shock, 12 new coronary revascularizations (4 IRA re-do angioplasty for restenosis or subacute stent thrombosis and 8 non-IRA new PCI for restenosis or *de novo* lesions). Only 8 (3.3%) patients reported pre-defined primary endpoint events.

IMR measured in the culprit coronary artery (both post-PPCI and at staged procedure) was not associated with the pre-defined clinical endpoint, regardless of the IMR cut-off value used for the analysis (median cut-off: IMR post-PPCI *p* = 0.281, IMR at staged procedure *p* = 0.446; cut-off value of 32: IMR post-PPCI *p* = 0.072, IMR at staged procedure *p* = 0.251). The results were unchanged even by adopting an IMR cut-off of 40 (IMR post-PPCI *p* = 0.279, IMR at staged procedure *p* = 0.99). Hence, we were not able to identify an IMR cut-off value to best predict primary endpoint events.

**FIGURE 2:**
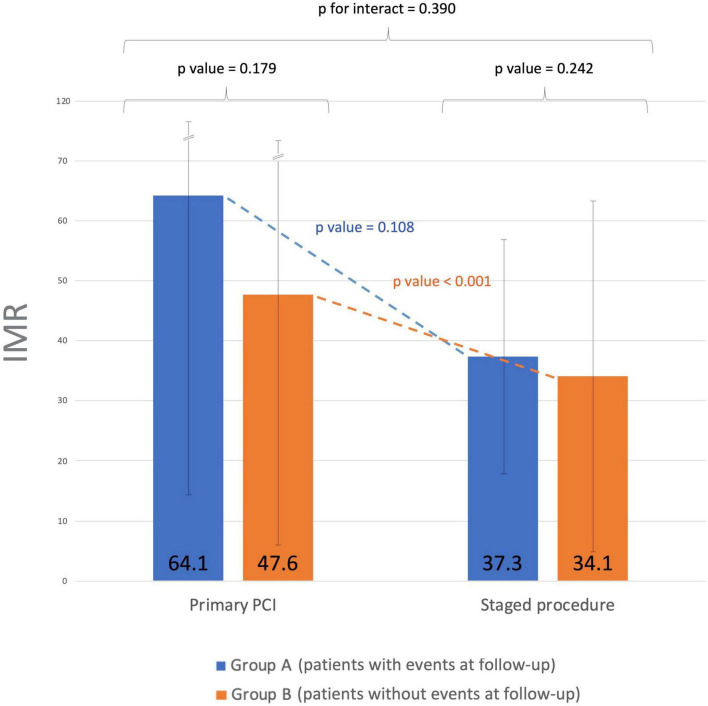
Index of microvascular resistance (IMR) time course from primary to staged procedure. Group A: patients who experienced primary endpoints. Group B: primary endpoints event-free patients. No significant differences in IMR values were observed between the two groups after successful PPCI of the culprit lesion. The IRA IMR significantly improved in event-free patients between PPCI and staged procedure. IMR, index of microvascular resistance; PPCI, primary percutaneous coronary intervention; IRA, infarct-related artery.

IMR variation between patients who experienced primary endpoints (Group A) and primary endpoints event-free patients (Group B) was also analyzed (Figure 2). No significant differences in IRA IMR after PPCI were observed between the 2 groups (64.1 ± 49.9 vs. 47.6 ± 42.8; *p* = 0.18), although a trend toward higher IMR values in the first group was noted, suggesting a likely worse outcome in patients with greater microcirculatory dysfunction at presentation. While IMR did not significantly change between PPCI and staged procedure (from 64.1 ± 49.9 to 37.3 ± 19.3; *p* = 0.11) in group A, in event-free patients the IRA IMR significantly improved (from 47.6 ± 42.8 to 34.1 ± 28.8; *p* < 0.001). However, considering the “delta” IMR (i.e., the variation of the index over time) from PCI to staged procedure in both groups, no statistically significant differences were observed (*p* = 0.39).

### Echocardiographic findings

**FIGURE 3:**
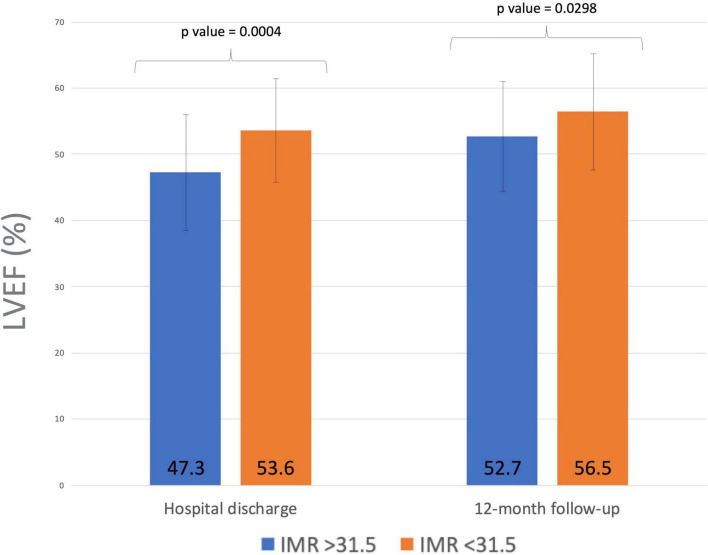
Index of microvascular resistance (IMR) after PPCI of the left anterior descending artery and left ventricular EF at hospital discharge and 12 months follow-up. Higher IMR measurements after PPCI predicted lack of improvement of LV function at follow-up in patients with anterior myocardial infarction. IMR, index of microvascular resistance; PPCI, primary percutaneous coronary intervention; EF, ejection fraction; LV, left ventricle.

Echocardiography evaluation of LV function was performed in all subjects before discharge and at 1-year follow-up. A significant improvement in LVEF (from 53.3 ± 8.6 to 56.2 ± 7.9%; *p* < 0.001) and a slight deterioration in LV wall motion score index (WMSI) (from 1.8 ± 2.9 to 1.9 ± 3.9; *p* < 0.001) were observed in the overall population. While LVEF improved from discharge to 1-year follow-up both in patients with high and low IMR, an improvement in WMSI (from 1.7 ± 2.4 to 1.5 ± 2.4, *p* < 0.0001) was observed only in patients with higher IMR values at presentation (Table 2). No relationship was found between the delta IMR (staged-primary) and changes in LV volumes and function in the whole study population. Notwithstanding, a correlation between median value of IMR and changes in LV function has been found in the subgroup of patients with anterior STEMI. Patients with an IMR >31.5 after PPCI of the LAD artery showed a lower LVEF value than those with a IMR < 31.5 both at hospital discharge and at 12 months follow-up (47.3 ± 8.4 vs. 53.6 ± 7.6; *p* = 0.0004 and 52.7 ± 7.7 vs. 56.5 ± 8.4; *p* = 0.0298; Figure 3).

**TABLE 2:** Echocardiographic measurements.

	Overall			IMR ≤32			IMR >32		
	Discharge	12 months	*P*-value (discharge vs. 12 months)	Discharge	12 months	*P*-value (discharge vs. 12 months)	Discharge	12 months	*P*-value (discharge vs. 12 months)
Ejection fraction (%)	53.3 ± 8.6	56.2 ± 7.9	<0.0001	54.5 ± 8.1	56.9 ± 8.2	0.0017	52.2 ± 8.9	55.6 ± 7.6	<0.0001
LVESV (ml)	50.6 ± 21.8	47.4 ± 20.3	0.0810	50.4 ± 19.8	46.8 ± 20.6	0.1514	50.8 ± 23.4	48.0 ± 20.1	0.2974
LVEDV (ml)	101.7 ± 28.5	102.3 ± 32.4	0.7884	103.5 ± 29.7	103.2 ± 33.8	0.4079	100.3 ± 27.5	101.6 ± 31.4	0.7251
LVESD (mm)	35.5 ± 8.5	33.9 ± 6.6	0.0038	34.4 ± 9.0	33.6 ± 6.4	0.2530	36.5 ± 7.9	34.3 ± 6.7	0.0023
LVEDD (mm)	51.4 ± 29.7	50.2 ± 7.5	0.3250	53.5 ± 41.6	50.6 ± 8.8	0.4079	49.3 ± 7.2	49.7 ± 6.0	0.5296
16 segment WMSI	1.8 ± 2.9	1.9 ± 3.9	<0.0001	1.9 ± 3.4	2.3 ± 5.1	0.0005	1.7 ± 2.4	1.5 ± 2.4	<0.0001

Values are mean ± SD (*n*). LVESV, left ventricular end systolic volume; LVEDV, left ventricular end diastolic volume; LVESD, left ventricular end systolic diameter; LVEDD, left ventricular end diastolic diameter; WMSI, wall motion score index.

## Discussion

The main findings of this prospective, multicenter, clinical study enrolling MVD patients undergoing PPCI for STEMI can be summarized as follows: (1) Coronary microvascular dysfunction, as assessed by IMR, is common in the infarct related territory and tends to recover early after revascularization; (2) The overall incidence rate of adverse events was unexpectedly low in our population at one-year follow-up; (3) No relationship between IRA-IMR, clinical events and LV remodeling was observed over the one-year follow-up period; (4) A correlation between IMR values and changes in LV function was found only in patients with anterior STEMI.

Despite PPCI in acute myocardial infarction can provide high epicardial coronary patency rates, insufficient myocardial reperfusion may occur as a result of microvascular obstruction and myocardial hemorrhage. Failure of reperfusion may have negative impact on outcome, being associated with LV adverse remodeling and dysfunction, re-hospitalization for heart failure and increased mortality (3). The IMR is a direct intravascular guidewire–based method for assessing coronary microvascular function on regional basis, which is quantitative in nature, and independent of the epicardial vessels size (14, 16).

In acute STEMI patients, coronary microvascular resistance as measured by IMR is usually increased. Conversely, coronary flow reserve (CFR), which reflects the vasodilatory potential of the coronary circulation, is significantly reduced. However, a progressive improvement of CFR and a reduction of IMR has been reported at 24 h and at 6 months after MI, indicating early and sustained recovery of microvascular function over time (25, 26). Our study confirms this finding, both in event-free patients and, to a lesser extent, in patients who experienced events at follow-up.

There is a large body of evidence showing that IMR measured immediately after PPCI is correlated with final infarct size as assessed by biomarker elevation, positron emission tomography, and CMR and predicts LV function at 3 and 6 months after STEMI (16, 24, 27–31). On the other hand, only few studies have focused on the value of IMR in predicting patient clinical outcome. Fearon et al. (17) assessed the incidence of death or re-hospitalization for HF in 253 single vessel disease patients undergoing PPCI for STEMI. During a median follow-up period of 2.8 years, 13.8% of the enrolled patients experienced the primary endpoint and 4.3% died. Patients with an IMR >40 had a higher risk of death or re-hospitalization for HF (hazard ratio [HR] 2.1; *p* = 0.034) and of death alone (HR 3.95; *p* = 0.028) at 1 year than patients with an IMR ≤40, while other indices of microvascular damage such as CFR were not predictive of clinical events. An IMR >40 was the only independent predictor of death alone (HR 4.3; *p* = 0.02) or re-hospitalization for HF at multivariable analysis. Similarly, in the study by Carrick et al. (18), 283 patients with STEMI were prospectively enrolled after PPCI and categorized according to IMR (≤40 or >40) and CFR (≤2.0 or >2) values. The primary endpoint of death or first CHF hospitalization occurred in 11% of patients during the index hospitalization or after discharge. An IMR >40 was associated with microvascular obstruction and negative changes in LVEF and LV end-diastolic volumes. Also, higher IMR values were associated with a 4-fold increase in all-cause death or heart failure at a median follow-up of 2.3 years. The combination of IMR with CFR did not have superior prognostic value. Scarsini et al. (19) reported comparable results in a group of 198 patients with STEMI undergoing PPCI. At long-term follow-up (mean follow-up of 40.1 months), patients with an IMR >40 and/or microvascular obstruction assessed by CMR reported worse clinical outcomes compared with those with lower IMR values and no microvascular obstruction.

Compared with these studies, our trial enrolled only patients with MVD at baseline angiography that were expected to be at higher risk of clinical adverse events at follow-up. However, we observed an unexpected low rate of adverse clinical events in our population and were not able to find a significant association between IMR and the pre-defined clinical outcome endpoints. As a matter of fact, the primary endpoint was met in only 3.3% of patients, and this may have led to a power issue in assessing differences in clinical outcome between patients according to the value of IMR. Possible explanations for the low event rate observed in the AMICRO trial may be a potential selection bias, the relatively short follow-up period and the strategy of revascularization, which included a staged evaluation of non-IRA lesions followed by FFR-guided complete revascularization (CR). Indeed, over the last few years, several studies have shown that in STEMI patients with MVD undergoing PPCI, a strategy of CR before hospital discharge may result in significant reduction of event rate at short term follow-up (32–37). In the current study, among 309 non-IRA lesions tested, appropriate revascularization was performed in the majority (90%) of lesions whose measured FFR was lower than 0.80. We can then speculate that in the present trial the observed event rate at follow-up may have been partially reduced by the effect of an FFR-guided CR strategy before hospital discharge.

In contrast to what we observed in the whole population, a correlation between IMR and changes in LV function was found in the subgroup of patients with anterior STEMI. Higher IMR measurements post-PPCI of the LAD artery were significantly associated with lower LVEF values both at hospital discharge and at 12-month follow-up. These results provide further support for the hypothesis that IMR value in predicting LVEF is most likely to be discriminative in patients with higher myocardium at risk such as those with large anterior infarction (29).

## Limitations

The low event rate observed at follow-up get the study unpowered to end-point. As already discussed, follow-up duration [shorter than the >2-year follow-up of other studies exploring the potential value of IMR in predicting patient clinical outcome (17–19)] and the strategy of revascularization may have contributed in part to the low event rate observed in the study. In addition, the short time frame from symptoms onset to revascularization, the low Killip class at admission, the relatively low rate of diabetes and the extensive use of radial approach may have further influenced to the low number of events observed. The absence of a screening log of the study population also does not allow for exclusion and definition of potential selection biases underneath. The low primary endpoint event rate may also explain the inability to identify an IMR cut-off value to best predict adverse events at follow-up, which was among the pre-specified secondary endpoints.

Non-homogeneous laboratory tests for myocardial damage were used (e.g., CK, Tn, and HsTn), making them difficult to use for clinical correlations. Also, it should be considered that the definition of STEMI has suffered numerous changes from 2013, when enrollment began.

The IMR measured after the PPCI was found to be predictive of lower EF values both at discharge and at 12 months follow-up only when the median IMR cut-off of 31.5 was adopted. Conversely, when using the pre-specified cut-off of 32, higher IMR values were associated with worse LVEF at discharge (53.5 ± 7.5 vs. 47.2 ± 8.5, *p* = 0.0004) but not at 12 months follow-up (56.2 ± 8.4 vs. 52.9 ± 7.7, *p* = 0.0586), although a trend was noted. In addition, we must recognize that more accurate echocardiographic parameters to determine subclinical LV dysfunction, such as speckle tracking, were not used in this study.

Furthermore, the study is limited by the lack of information on CFR even though, in patients with STEMI, IMR and not CFR demonstrated a significant association with clinical outcomes (18, 38). Data about Pd/Pa and hyperemic transit time were also not collected.

Lastly, although a close monitoring was performed during the entire follow-up to minimize the loss of patients, a total of 16 patients (6.6%) were lost to follow-up/withdrawn.

## Conclusion

Despite the routine success of PPCI in STEMI, impaired coronary microvascular function is commonly detected by IMR evaluation in the infarct territory and shows a slow recovery early over the time after reperfusion. Post-angioplasty IMR values negatively correlated with LVEF only in patients with anterior acute MI. Further work is required to establish the validity of IMR in predicting clinical outcomes in specific subgroups of patients.

## Data availability statement

The raw data supporting the conclusions of this article will be made available by the authors, without undue reservation.

## Ethics statement

The studies involving human participants were reviewed and approved by Comitato Etico Area Vasta Sud-Est (SI) Comitato Etico Provinciale dell’Insubria (VR) CESC province di Treviso e Belluno (TV) CESC della provincia di Padova (PD) Comitato Etico Palermo 2 (PA) Comitato Etico Regionale Liguria (GE) Comitato Etico dell’Area Vasta Emilia Nord (RE) Comitato Etico Interregionale (BA) Comitato Etico Regionale delle Marche (AN) Comitato Etico referente per l’Area di Pavia (PV). The patients/participants provided their written informed consent to participate in this study.

## Author contributions

MF, EV, GTa, AM, KL, and LU contributed to the study design. All authors contributed to patient enrolment/management and/or data analysis/article writing, and all have approved the final manuscript.

## Supplementary material

Supplementary Figure 1 Treatment of infarct related vessel (IRA) lesions and non-infarct related vessel (non-IRA) lesions.

## Abbreviations

CABG: coronary artery bypass graft
CFR: coronary flow reserve
CHF: congestive heart failure
CMR: cardiac magnetic resonance
CR: complete revascularization
EF: ejection fraction
FFR: fractional flow reserve
FSS: functional syntax score
HF: heart failure
ICD: implantable cardioverter defibrillator
IMR: index of microvascular resistance
IRA: infarct-related artery
LAD: left anterior descending artery
LV: left ventricle
MI: myocardial infarction
MVD: multivessel disease
PCI: percutaneous coronary intervention
PPCI: primary percutaneous coronary intervention
SS: syntax score
STEMI: ST-elevation myocardial infarction
TIMI: thrombolysis in myocardial infarction
WMSI: wall motion score index.
