# Supplementary figures and images for: One-year results from the Assessing MICRO-vascular resistances via IMR to predict outcome in ST-elevation myocardial infarction patients with multivessel disease undergoing primary PCI (AMICRO) trial
Source: Front Cardiovasc Med. 2022 Dec 2;9:1051174. doi: 10.3389/fcvm.2022.1051174 (PMC9755670; doi:10.3389/fcvm.2022.1051174)

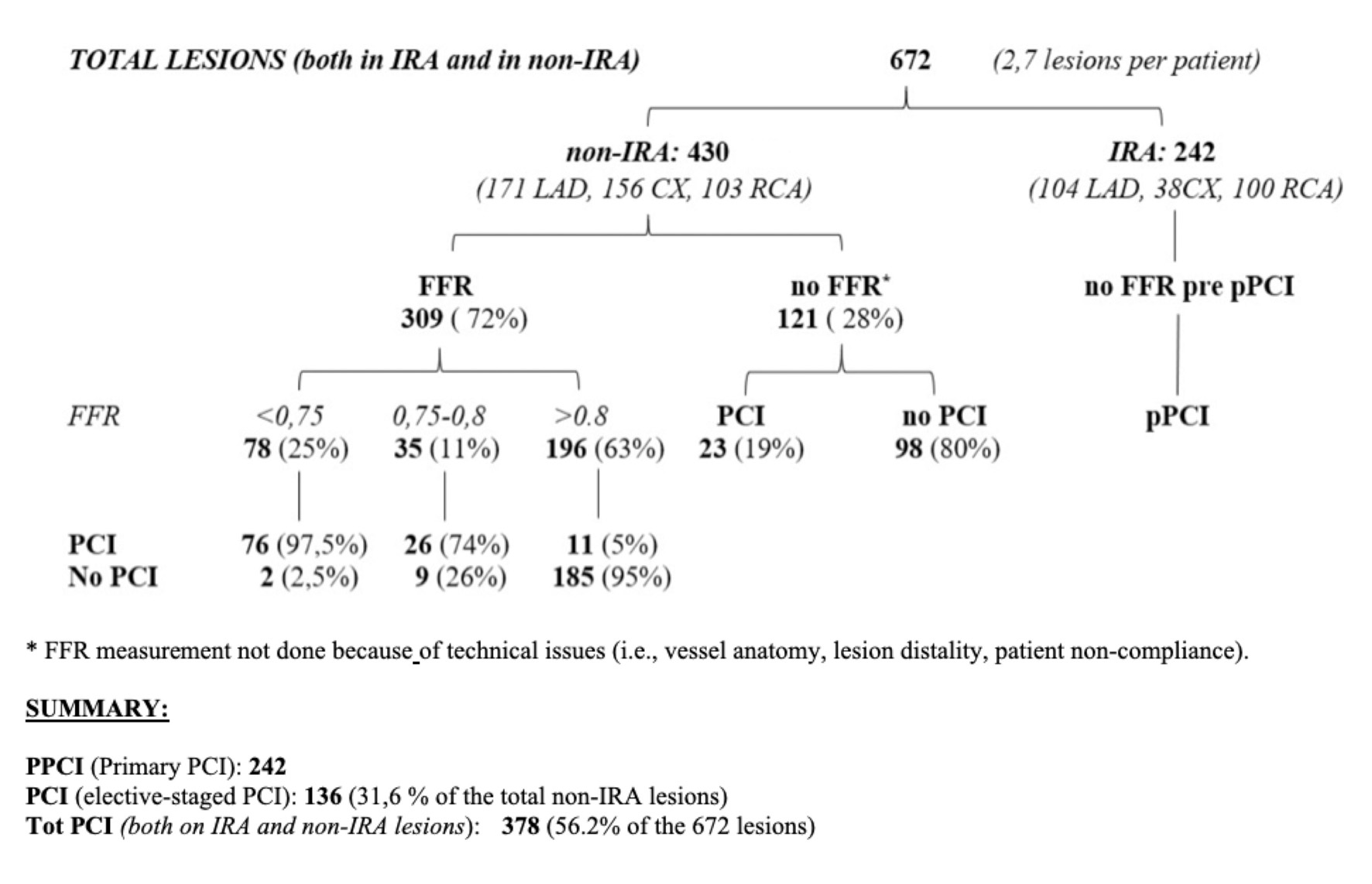

Supplement: Supplementary Figure 1 — Treatment of infarct related vessel (IRA) lesions and non-infarct related vessel (non-IRA) lesions. [file Image_1.JPEG]
